# Supplementary material for: A method for comparing MRI sequences of the knee for segmentation based on morphological features
Source: PLoS One. 2024 Dec 27;19(12):e0311532. doi: 10.1371/journal.pone.0311532 (PMC11676894; doi:10.1371/journal.pone.0311532)
Supplement: S1 Table — (DOCX) [file pone.0311532.s001.docx]

**Supplementary Table 1.** Results of the edge sharpness and contrast: femur

| Subject | Sequence | Sharpness | | | | | Contrast | | | | |
| --- | --- | --- | --- | --- | --- | --- | --- | --- | --- | --- | --- |
|  |  | E_BB_ | E_BC_ | E_CF_ | E_CM_ | E_BT_ | E_BB_ | E_BC_ | E_CF_ | E_CM_ | E_BT_ |
| 1 | T1 | 35.13 | 10.85 | 48.09 | 23.14 | 26.28 | 3.19 | 1.84 | 1.84 | 2.13 | 3.43 |
|  | PD | 5.90 | 8.88 | 18.26 | 21.28 | 7.06 | 1.65 | 3.42 | 1.79 | 2.40 | 2.71 |
|  | SPGR | 2.39 | 24.73 | 23.70 | 11.56 | 2.94 | 1.43 | 4.07 | 3.72 | 1.32 | 1.87 |
| 2 | T1 | 64.41 | 11.99 | 71.60 | 18.82 | 43.60 | 4.46 | 1.78 | 2.11 | 2.70 | 5.79 |
|  | PD | 6.14 | 13.44 | 22.29 | 18.27 | 5.06 | 2.15 | 3.27 | 1.65 | 2.85 | 2.85 |
|  | SPGR | 3.85 | 24.98 | 26.37 | 4.14 | 3.50 | 1.71 | 4.96 | 4.16 | 1.25 | 1.95 |
| 3 | T1 | 33.50 | 13.36 | 32.18 | 34.05 | 23.33 | 2.64 | 1.81 | 1.74 | 2.84 | 4.26 |
|  | PD | 3.35 | 10.42 | 15.72 | 31.19 | 4.67 | 1.48 | 4.42 | 1.60 | 4.03 | 2.62 |
|  | SPGR | 2.79 | 20.06 | 19.37 | 7.11 | 6.91 | 1.37 | 3.80 | 3.12 | 1.27 | 3.02 |
| 4 | T1 | 41.08 | 7.50 | 25.85 | 32.57 | 27.77 | 3.04 | 1.61 | 1.58 | 2.56 | 4.07 |
|  | PD | 3.59 | 6.87 | 10.69 | 30.18 | 3.19 | 1.34 | 4.03 | 1.30 | 2.49 | 2.77 |
|  | SPGR | 2.97 | 20.38 | 27.83 | 5.06 | 5.21 | 1.44 | 4.61 | 5.15 | 1.08 | 2.73 |
| 5 | T1 | 47.70 | 19.94 | 35.04 | 24.78 | 31.15 | 4.23 | 2.38 | 1.52 | 1.92 | 4.40 |
|  | PD | 8.15 | 20.99 | 35.92 | 47.77 | 11.99 | 2.02 | 4.42 | 1.63 | 2.83 | 3.70 |
|  | SPGR | 6.29 | 6.32 | 16.45 | 6.69 | 6.55 | 2.21 | 2.43 | 1.57 | 1.06 | 3.30 |
| 6 | T1 | 50.27 | 21.86 | 30.29 | 20.72 | 42.81 | 3.93 | 2.14 | 1.64 | 2.08 | 5.09 |
|  | PD | 7.07 | 16.37 | 17.20 | 33.07 | 4.73 | 2.23 | 5.83 | 1.22 | 2.93 | 2.39 |
|  | SPGR | 4.75 | 16.48 | 21.33 | 4.93 | 3.92 | 1.70 | 3.45 | 1.78 | 1.16 | 2.27 |
| 7 | T1 | 31.12 | 6.47 | 18.34 | 13.95 | 19.96 | 2.84 | 1.67 | 1.38 | 1.92 | 2.99 |
|  | PD | 5.68 | 7.36 | 21.41 | 51.16 | 5.29 | 1.60 | 3.42 | 1.46 | 2.02 | 2.19 |
|  | SPGR | 4.08 | 19.15 | 12.55 | 4.35 | 3.70 | 1.85 | 3.29 | 1.59 | 1.13 | 2.03 |
| 8 | T1 | 40.50 | 9.86 | 35.86 | 27.57 | 39.71 | 3.70 | 2.48 | 1.59 | 2.45 | 4.83 |
|  | PD | 7.83 | 11.35 | 22.55 | 49.37 | 11.50 | 1.96 | 4.43 | 1.31 | 6.71 | 3.69 |
|  | SPGR | 4.55 | 28.42 | 19.28 | 4.66 | 5.45 | 1.61 | 4.17 | 2.69 | 1.30 | 2.09 |
| 9 | T1 | 53.97 | 11.46 | 33.74 | 30.64 | 43.33 | 3.84 | 2.16 | 1.60 | 2.52 | 4.21 |
|  | PD | 13.75 | 13.82 | 17.95 | 42.00 | 6.30 | 2.77 | 4.51 | 1.63 | 3.65 | 2.18 |
|  | SPGR | 2.83 | 25.74 | 16.77 | 8.86 | 2.74 | 1.48 | 4.74 | 2.32 | 1.29 | 1.56 |
| 10 | T1 | 54.62 | 20.08 | 55.61 | 23.94 | 33.58 | 4.09 | 2.03 | 1.70 | 1.96 | 4.76 |
|  | PD | 9.73 | 14.87 | 11.45 | 28.90 | 8.39 | 2.55 | 4.75 | 1.49 | 2.40 | 3.21 |
|  | SPGR | 6.01 | 27.01 | 21.00 | 3.50 | 5.03 | 1.92 | 3.85 | 1.82 | 1.15 | 2.55 |
| 11 | T1 | 46.69 | 9.71 | 64.40 | 22.70 | 25.86 | 3.13 | 1.58 | 2.08 | 1.98 | 3.86 |
|  | PD | 4.85 | 9.02 | 20.83 | 48.69 | 4.37 | 1.60 | 3.41 | 1.82 | 3.10 | 2.59 |
|  | SPGR | 3.85 | 29.43 | 33.40 | 6.64 | 3.98 | 1.49 | 4.55 | 4.52 | 1.27 | 1.99 |

Note.-T1 = T1-weighted, PD = proton density-weighted, SPGR = spoiled gradient-echo, E_BB_: edge between cancellous bone and cortical bone, E_BC_: edge between cortical bone and cartilage, E_CF_: edge between cartilage and fat, E_CM_: edge between cartilage and meniscus, E_BT_: edge between cortical bone and tissue
