# Supplementary material for: A method for comparing MRI sequences of the knee for segmentation based on morphological features
Source: PLoS One. 2024 Dec 27;19(12):e0311532. doi: 10.1371/journal.pone.0311532 (PMC11676894; doi:10.1371/journal.pone.0311532)
Supplement: S2 Table — (DOCX) [file pone.0311532.s002.docx]

**Supplementary Table 2.** Results of the edge sharpness and contrast: tibia

| Subject | Sequence | Sharpness | | | | Contrast | | | |
| --- | --- | --- | --- | --- | --- | --- | --- | --- | --- |
|  |  | E_BB_ | E_BC_ | E_CM_ | E_BT_ | E_BB_ | E_BC_ | E_CM_ | E_BT_ |
| 1 | T1 | 43.05 | 5.89 | 29.34 | 27.41 | 3.22 | 1.94 | 2.35 | 3.50 |
|  | PD | 5.67 | 10.21 | 38.85 | 9.49 | 1.44 | 2.49 | 2.87 | 2.54 |
|  | SPGR | 3.03 | 20.80 | 16.02 | 6.82 | 1.67 | 5.03 | 1.55 | 2.01 |
| 2 | T1 | 62.96 | 6.88 | 25.15 | 45.92 | 4.34 | 1.84 | 2.36 | 4.87 |
|  | PD | 7.82 | 10.77 | 27.88 | 5.86 | 2.43 | 3.10 | 2.91 | 2.86 |
|  | SPGR | 4.19 | 26.35 | 4.64 | 3.33 | 1.77 | 5.16 | 1.25 | 1.77 |
| 3 | T1 | 40.09 | 5.72 | 37.92 | 32.23 | 3.51 | 1.36 | 2.99 | 4.08 |
|  | PD | 4.03 | 8.20 | 32.21 | 5.26 | 1.69 | 4.10 | 4.58 | 2.72 |
|  | SPGR | 2.57 | 21.31 | 8.33 | 8.96 | 1.31 | 4.29 | 1.31 | 3.56 |
| 4 | T1 | 40.52 | 7.38 | 32.06 | 31.94 | 2.75 | 1.62 | 2.57 | 3.89 |
|  | PD | 4.09 | 6.73 | 30.34 | 3.11 | 1.40 | 3.99 | 2.60 | 2.92 |
|  | SPGR | 3.07 | 19.54 | 4.67 | 4.59 | 1.38 | 4.64 | 1.06 | 2.56 |
| 5 | T1 | 50.10 | 18.91 | 24.82 | 33.30 | 4.01 | 2.38 | 1.91 | 4.55 |
|  | PD | 8.53 | 21.82 | 48.49 | 9.74 | 2.08 | 4.57 | 2.80 | 3.47 |
|  | SPGR | 5.85 | 5.46 | 6.86 | 6.68 | 2.29 | 2.29 | 1.06 | 3.41 |
| 6 | T1 | 48.46 | 22.84 | 25.58 | 42.41 | 4.17 | 2.32 | 2.15 | 5.62 |
|  | PD | 7.25 | 16.76 | 34.34 | 4.78 | 2.24 | 8.14 | 3.07 | 2.51 |
|  | SPGR | 5.19 | 17.26 | 5.81 | 4.30 | 1.69 | 3.93 | 1.18 | 2.31 |
| 7 | T1 | 35.13 | 6.38 | 12.98 | 25.84 | 3.06 | 1.69 | 1.89 | 3.16 |
|  | PD | 5.99 | 6.93 | 51.16 | 4.97 | 1.48 | 3.39 | 2.02 | 2.04 |
|  | SPGR | 3.59 | 19.17 | 4.01 | 3.47 | 1.67 | 3.13 | 1.12 | 1.89 |
| 8 | T1 | 46.18 | 8.88 | 28.69 | 42.10 | 3.56 | 2.83 | 2.43 | 4.48 |
|  | PD | 6.94 | 11.15 | 49.01 | 11.67 | 1.75 | 4.67 | 6.42 | 3.55 |
|  | SPGR | 4.08 | 28.02 | 4.17 | 5.47 | 1.55 | 5.12 | 1.26 | 2.03 |
| 9 | T1 | 57.65 | 12.46 | 31.98 | 48.40 | 3.89 | 2.40 | 2.54 | 4.08 |
|  | PD | 15.49 | 13.76 | 43.63 | 5.90 | 2.71 | 4.41 | 3.73 | 2.15 |
|  | SPGR | 2.77 | 29.14 | 9.97 | 2.81 | 1.49 | 5.22 | 1.30 | 1.40 |
| 10 | T1 | 57.43 | 18.54 | 25.22 | 33.08 | 3.81 | 2.08 | 1.85 | 4.27 |
|  | PD | 9.69 | 18.29 | 30.90 | 8.64 | 2.54 | 5.29 | 2.58 | 3.00 |
|  | SPGR | 6.25 | 25.27 | 3.31 | 4.67 | 1.72 | 3.69 | 1.14 | 2.41 |
| 11 | T1 | 48.53 | 10.09 | 23.05 | 29.70 | 3.18 | 1.62 | 1.96 | 3.65 |
|  | PD | 5.08 | 10.98 | 49.31 | 4.85 | 1.62 | 3.61 | 3.04 | 2.74 |
|  | SPGR | 4.00 | 27.63 | 5.32 | 4.37 | 1.48 | 4.19 | 1.21 | 1.92 |

Note.-T1 = T1-weighted, PD = proton density-weighted, SPGR = spoiled gradient-echo, E_BB_: edge between cancellous bone and cortical bone, E_BC_: edge between cortical bone and cartilage, E_CF_: edge between cartilage and fat, E_CM_: edge between cartilage and meniscus, E_BT_: edge between cortical bone and tissue
