# Supplementary material for: A method for comparing MRI sequences of the knee for segmentation based on morphological features
Source: PLoS One. 2024 Dec 27;19(12):e0311532. doi: 10.1371/journal.pone.0311532 (PMC11676894; doi:10.1371/journal.pone.0311532)
Supplement: Attachment — Submitted filename: Response to reveiwers_1st revision.docx [file pone.0311532.s003.docx]

We thank the Reviewers for the time and effort that has gone into providing constructive criticism of our manuscript. We have revised the manuscript according to all the Reviewers' requests which we believe have improved the manuscript significantly.

In the following, we present our point-by-point responses to the Reviewers' comments. Parts written in *italic with underline* are new text added to the manuscript.

**Reviewer 1**

R1-1. Remove all typos from the text. The abstract should be improved (conclusions in the abstract!) The Nomenclature part should be added for all abbreviations and symbols used in the text.
→ Thank you for makirng us aware of this. We have corrected typos and added Nomenclatures. Also, conclusion part in the abstract was changed so it now reads:

*The ultimate goal of this study is to present a methodology for selecting the most appropriate MRI sequence for segmentation, which can be applied to images of other parts in addition to the knee images used in the study. The method we present quantitatively evaluates the edge characteristics, and experimental results show that our method shows consistent results according to the edge. Our method will provide additional information for MRI sequence selection for segmentation.*

R1-2. What is the limitations of the used technique?

→ Thank you for the question. Our method requires manual (by human) marking to define edges. In ambiguous edges, there will be differences in edge markings by different people, and this difference will lead to differences in results. This will be a limitation of our proposed technique. We added this limitation in the Discussion section.

*Our method requires manual (by human) marking to define edges. In ambiguous edges, there will be differences in edge markings by different people, and this difference will lead to differences in results. This will be a limitation of our proposed technique. Therefore* *selecting areas with relatively clear edges will be necessary for practical use* x

R1-3. Add references to the equations used!

→ We have added reference [22] for Eq. 1. Eq 2-5 are the formula we defined.

*22. Lynn H, Shlomo Z. Advanced Calculus. Revised ed. Jones and Bartlett Publishers; 1990.*

R1-4. Add more details to the proposed image processing algorithm.

→ Thanks for this comment. The two metrics (sharpness and contrast) were computed using a profile (virtual ray) that has image pixel information. Except interpolation process of profile, we did not directly process the image information. The signal processing step of the profile is expressed in “Quantitative analysis of edge using virtual ray” section and Fig. 2-3. We also added the algorithm flow for intuitive understanding.

*The overall process for sequence evaluation at edges defined by manual marking consists of the following steps. (1) Computing of the unit normal vectors, (2) Virtual ray propagation, (3) Extraction of pixel profile from virtual ray, (4) Profile interpolation, (5) Computing a sharpness and contrast.*

R1-5. Enrich the introduction part with recent articles on Otsu’s thresholding technique for MRI image brain tumor segmentation.

→ Thank you for the suggestions. This study is about how to select MRI sequences for the best segmentation performance. Our introduction explains this. Article “Otsu’s thresholding technique for MRI image brain tumor segmentation” is about a specific segmentation method , which is not relevant for our specific study.

R1-6. The discussion part should be improved.

→ Thank you for your advice I have corrected this in all Discussion section.

*Finally, our method requires manual (by human) marking to define edges. In ambiguous edges, there will be differences in edge markings by different people, and this difference will lead to differences in results. This will be a limitation of our proposed technique. Therefore,* *selecting areas with relatively clear edges will be necessary for practical use.*

*Also, this study was conducted using data from healthy subjects without any specific knee pathology. In general, changes in tissue characteristics due to disease reduce the segmentation performance. This effect may be a larger variable than the difference in sequences. However, only a small amount of normal data was used since the purpose of this study was to present the methodology rather than to find the best sequence for a specific region.*

R1-7. Write the conclusion part in the form of important points.

→ Thank you for your suggestion. We have corrected this.

*Currently, MRI sequences vary greatly depending on equipment manufacturers, and there are hundreds of sequences, including those developed for research purposes. However, this study was conducted with only three sequences. Therefore, the results presented in this study will not be the best sequence for knee segmentation. Our purpose in this study was to present a new method to evaluate the edge sharpness and contract to enable, for instance, determining the best-suited sequences for segmentation. In conclusion, our new method can help quantitatively compare image sharpness and contrasts from different MRI sequences for the segmentation algorithm. The proposed method can be used to aid in selecting appropriate MRI sequences for segmentation algorithms.*

**Reviewer 2**

The authors of the study aim to analyze a new method for automatic/semi-automatic segmentation of structures in magnetic resonance imaging (in this case, the knee, but with the possibility of extending to other regions or structures). Three types of sequences were chosen: T1, PD, and SPGR, and five kinds of edges between structures (cortical bone-cancelous bone, cortical bone-cartilage, cartilage-fat, cartilage-meniscus, cortical bone-tissue). The authors explain why two features - edge sharpness and contrast between two different types of tissue—are so important for segmentation methods (semi-automatic or using machine learning). The authors do not intend to decide which is the best sequence for diagnosis or segmentation of a specific structure, but to provide a tool to decide, depending on the structure we need to segment, which sequence would be the best, as clearly outlined in the discussion chapter. The authors explain in detail the image acquisition technique on healthy volunteers using standard practice MRI sequences. The usual parameters of these sequences are explained, specifically in what types of pathologies they can be useful. Using elaborate mathematical methods, after manually tracing the edges, the authors calculate contrast and edge sharpness indices to decide which sequence is more useful for which type of segmentation. The authors highlight the difficulty of tracing the edges of structures in the human body, a process different from tracing edges between other types of materials, and explain why manual (human) tracing was chosen, followed by computerized analysis of this human tracing. The authors acknowledge limitations of the study, such as the different slice thickness between the three types of sequences, which may influence the analysis. However, the study aims to establish a method of analysis, not to provide the answer to which sequence is the best. One of the important conclusions is that none of the sequences is perfect for any type of structure we want to segment. It should be noted that the study aims to explain the use of a new analysis method to serve as a decision tool for choosing the best sequence concerning the structure we want to segment (cartilage, cortical bone, spongy bone, etc.). The study has a clear, well-explained methodology, and the conclusions are well-defined. For radiologists and imaging researchers this study provides a tool that might prove to be very useful.

→ We deeply appreciate your deep understanding of the purpose and methodology of this study. We also appreciate your understanding of the reasons for the limitations (different slice thickness) of this study that we have mentioned. Yes, our study is not an evaluation of a specific area, but rather provides an overall methodology.

R2-1. Lines 220-226 suggestion: The information is quite complex; it might be useful to synthesize it in a table: for edge sharpness and for contrast - with each sequence and the conclusion or which sequence was the best for which type of edge detection.
→ Thanks for your suggestion. We also think this part is a bit complicated. However, this part is the conclusion derived from tables 2 and 3. We think that a new table for this is will be a duplication of Tables 2 and 3 and have therefore omitted to add a new table.

R2-2. Although it may not be important given the purpose of using a new segmentation method, the fact that healthy subjects without particular knee pathologies were used could be one of the study's limitations and maybe mention this in limitations.

→ Thank you for the comment we agree and haveadded this part in end of discussion section.

*Finally, this study was conducted using data from healthy subjects without any specific knee pathology. In general, changes in tissue characteristics due to disease reduces the segmentation performance. This effect may be a larger variable than the difference in sequences. However, only a small amount of normal data was used since the purpose of this study is to present the methodology rather than to find the best sequence for a specific region.*

R2-3. Line 148 proton density-weighted (PD) - there is an imaging inconsistency: images shown as an example - line 376 - the image annotated as PD is in fact “PD with fat saturation”. This propagates throughout the article and deserves further clarification: is the sequence tested actually PD or PD with fat saturation?

→ Thanks for pointing out our mistake. We used the proton density fat-saturated (PDFS). We modified all throughout the manuscript.

R2-4. Lines 152-154 - there might be a slight misunderstanding - on SPGR sequences, bone marrow is difficult to interpret, and it certainly does not come into direct contact with the cartilage (the cartilage is separated from the bone marrow by the cortical bone).

→ Thank you for making us aware of this. We have modified the text accordingly.

*Finally, the SPGR is known to have good contrast between cartilage and surrounding bone through fat suppression and has a high contrast-to-noise ratio.*

R2-5. Lines 121 to 123 are a bit confusing, maybe that phrase can be reformulated.

→ Thank you for thesuggestion. We modified the sentences.

*Typically, segmentation algorithms are developed and tuned using a specific sequence. Therefore, it may not be a good idea to use the performance of automatic segmentation to determine the best sequence. This is because the sequence used in the development phase is likely to show high performance.*

R2-6. Line 124 - “materials” refers in the human body as “tissue,” and maybe the use of “tissue” or “tissular” could be a more appropriate term when referring to human MRI images.

→ Thank you for making us aware of this. The “materials” replaced to “tissue”.

**Reviewer 3**

R3-1. The sequences are not fully named in the text and in the fig. 1. Thus: the first 2 sequences are annotated with the weighting type of (T1 and PD), and the third one, with the type of sequence (SPGR). Both annotations must be made (respectively T1 FSE/SE, PD FSE/SE and 3D (probably) T1 FSPGR. In addition, both the PD sequence and the FSPGR are fat saturation acquisitions, which should also be noted in the text and on the image.
→ Thank you for making us aware of this issue. We modified all annotations in the manuscript.

R3-2. Was the data tested for normality? Was ANOVA the most appropriate test or was a nonparametric test needed?

→ We tested for normality using Kolmogorov-Smirnov (p>0.05). We used parametric tests since the defined metrics (sharpness and contrast) showed normal distributions at each edge. The independent variable is a “sequence” in the five edges evaluated, so we used ANOVA.

*The normality was tested using Kolmogorov-Smirnov (p > .05).*

R3-3. The big difference in the acquisition parameters of the sequences used for comparison and the small number of patients included in the study are the major limitations of this study. From these two points of view, I think that the study could be improved.

We agree with the reviewer that using more sequences and more patient data would make for a richer study. However, our study aims to provide an overall methodology rather than evaluating a specific body part. So, to demonstrate the proposed method, we believe that the sample size is sufficiently.
